# Focal Vibration Alters Human Digital Sensory Nerve Action Potentials: A Pilot Study

**DOI:** 10.1155/2021/8819169

**Published:** 2021-03-03

**Authors:** Dong Qing Zhu, Fang Liu, Yu Zhu, Duan Lei, Xiang Jin, Lan Xu, Chao Jun Zheng, Robert Weber, Xiang Jun Chen

**Affiliations:** ^1^Department of Neurology, Huashan Hospital, Fudan University, Shanghai, China; ^2^Department of Rehabilitation Medicine, The Second Affiliated Hospital of Jiaxing University, Jiaxing, China; ^3^Department of Physical Medicine and Rehabilitation, Upstate Medical University Hospital, Syracuse, USA; ^4^Department of Traumatology and Orthopedics, Yueyang Hospital, Shanghai University of Traditional Chinese Medicine, Shanghai, China; ^5^Department of Orthopedic Surgery, Huashan Hospital, Fudan University, Shanghai, China

## Abstract

**Introduction:**

We studied the impact of vibratory stimulation on the electrophysiological features of digital sensory nerve action potential (SNAP).

**Methods:**

The antidromic digit 3 SNAP was recorded in 19 healthy adults before, during, and after applying a vibration to either 3rd or 5th metacarpal phalangeal joint (MCPJ) at 60 Hz and amplitude of 2 mm. 100% supramaximal stimulus intensity was performed in 5 subjects (randomly selected from the 19 subjects) where the SNAP sizes were recorded.

**Results:**

The amplitude of digit 3 SNAP declined to 58.9 ± 8.6% when a vibration was applied to MCPJ digit 3. These impacts did not change by increasing the electrical stimulus intensity. The SNAP regained its baseline value immediately after the cessation of vibration stimulation. The magnitude of size reduction of digit 3 SNAP was less when vibration was moved to from MCPJ of digit 3 to MCPJ of digit 5. *Discussion*. The marked drop of the SNAP size during vibratory stimulation reflects the decreased responsiveness of A*β* afferents to electrical stimulation, which deserve further investigation in the study of focal vibration in neurorehabilitation.

## 1. Introduction

Vibration exerts powerful stimulation on neuromuscular system. Numerous studies have confirmed the effects of focal vibratory stimuli at various levels of the nervous system and the therapeutic effects of focal vibration in neurorehabilitation for patients with motor and sensory impairment conditions such as stroke, spinal cord injury, multiple sclerosis, Parkinson's disease and dystonia [1–3]. When applied to the human hands, vibration activates the cutaneous mechanoreceptors, including Merkel disk for low frequencies (5–15 Hz), Meissner's corpuscles for midrange (20-50 Hz) in the superficial layers of the skin, and Pacinian corpuscles for high frequencies (60-400 Hz) in deeper layers of the skin and periosteum [4–6]. These receptors are innervated by A*β*-type myelinated fibers.

The main component of the digital sensory nerve action potential (SNAP) is produced by the summation of action potentials of large, myelinated A*β* fibers. The size of SNAP is proportional to the number of nerve axons depolarized by the testing electrical stimulation. Both the function of skin mechanoreceptors and A*β* fibers during vibration could affect the measures of digital 3 SNAP. Our study was designed to examine the electrophysiological features of digital SNAP during acute and transient exposure to vibratory stimulation.

## 2. Methods

### 2.1. Subjects

**Figure 1:**
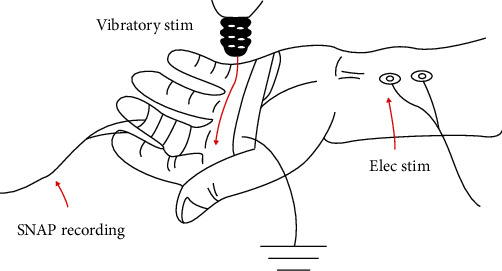
Schematic diagrams illustrating the settings of the experiment with conditioning stimulation of vibration.

Nineteen healthy subjects (10 men), aged 23-50 (mean, 32) years, with no known neuromuscular or musculoskeletal disorders participated in this study. They were recruited from the university research center population. The Human Ethics Committee of Huashan Hospital, Fudan University, China, granted the ethical committee approval, and each subject gave his/her informed consent prior to the study. Each subject was seated comfortably on a chair, with the left forearm and hand supinated on a solid wooden table with the fingers relaxed and unsupported (Figure 1).

Only minimal discomfort was caused by the brief application of vibration to the palm either at metacarpophalangeal joint (MCPJ) of digit 3 or MCPJ of digit 5. No visible venous stasis or color changes on the fingers were observed on any subject.

### 2.2. Stimuli

The median nerve was stimulated with a bar electrode at the midwrist 13 cm proximal to the active recording electrode. The electrical stimulation consisted of a square wave, 0.1 ms in duration, and was delivered at a rate of 2/s. The stimulus intensity started at a level below the threshold of the action potential and was incrementally increased until the maximal response was reached. The intensity was then increased by an additional 20% to ensure the supramaximal activation of SNAP.

With five subjects, we used 100% supramaximal stimulus to test the effects of an additional 100% increase in intensity for a maximal achievable activation of the sensory axons.

### 2.3. Recording

The antidromic median nerve SNAPs were recorded from the left hand with a self-adhesive ring electrode (Nihon Kohden, MEB-9400, Japan) placed 1 cm distal to the metacarpophalangeal joint (MCPJ) of the digit 3 with the reference electrode 4 cm further distally. A surface ground was adhered to the skin between the stimulating and recording electrodes. The impedance was maintained below 5 k ohm.

### 2.4. Palm Vibration

We utilized a hand-held massage vibrator (YH-3U, Yihe Electronic, China) with the vibration frequency at 60 Hz and a displacement of 2 mm. The vibration was applied to the palm at the MCPJ of digit 3. A constant force was applied to the MCPJ by using the own weight of the vibrator (0.9 kg). The diameter of the circular contact area between the skin and the vibrator was 2.5 cm. The vibrator was secured manually rather than being strapped to the palm. This method worked better in keeping the vibrator in place and ensuring the constant force of application during the experiment.

In addition, the digit 3 SNAP was recorded with the vibrator applied to the palm at MCPJ of digit 5 for seven subjects.

### 2.5. SNAPs Were Recorded in the following Steps

1. Before the start of vibration—as the baseline
2. During continuous vibration—register 20 SNAPs
3. After the cessation of the vibratory stimuli

Measurements of SNAPs included (1) amplitudes from the baseline to the negative peak and (2) onset latencies. The digital skin temperature was maintained at 32 ± 0.5°C.

### 2.6. Statistical Analysis

Statistical evaluation was performed by Student's *t*-test for paired data. Values, given as mean ± SD, were considered significant at *P* < 0.05.

## 3. Results

The traces of SNAP recorded before, during, and after vibration showed a satisfactory signal to noise ratio, without increased noise from muscle activity or electromagnetic interferences during vibration.

### 3.1. Vibration Stimulation at MCPJ 3

The amplitude of digit 3 SNAP showed a significant decrease (*P* < 0.05) from the baseline value during vibration (Table 1, Supplementary Figure 1, Supplementary Table 1). The use of 100% supramaximal intensity did not produce a regaining of the amplitude of SNAP. The amplitude of the SNAP reverted back to the baseline level immediately when the vibration stimulation ceased in both cases. The mean onset latency remained unchanged before, during, and after vibration.

### 3.2. Vibration Relocated to the MCPJ Digit 5

**Figure 2:**
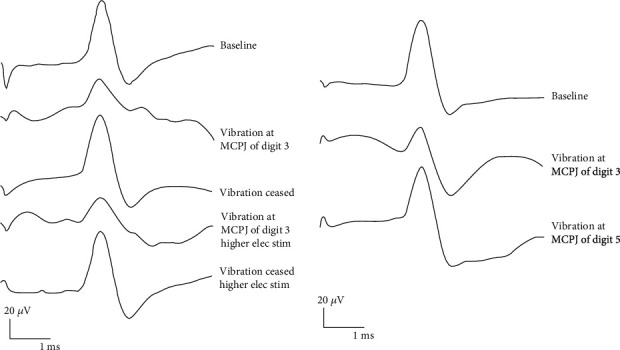
Digit 3 SNAP. (a) Recordings from subject 14, the amplitude of SNAP reduces with the similar extent (52.4.0% vs. 54.1%) at the two different stimulation intensities. The SNAP amplitude regains its baseline value after vibration. (b) Recordings from subject 3, illustrating a smaller reduction of digit 3 SNAP amplitude when vibration applied to the MCPJ of digit 5 (62.9%) in comparison with the greater reduction when vibration to the MCPJ of digit 3 (31.5%). No notable changes of the onset latencies during or after vibration.

In seven subjects, when the vibration was moved to MCPJ of digit 5, the reduction of the amplitude of digit 3 SNAP was significantly smaller than when the vibration was applied to the MCPJ of digit 3 (*P* < 0.05 (Table 1). The SNAP amplitude again regained its baseline value after vibration ceased. There was no notable change in the onset latency of the SNAP throughout the experiments (Figure 2).

## 4. Discussion

We found that the mechanical vibration applied to the palm remarkably reduces the size of the digital SNAP. In addition, the SNAP amplitude returned to the baseline level immediately after the cessation of the vibration.

The fact that the digit 3 SNAP reduction was smaller when the vibration was moved from MCPJ of digit 3 to digit 5 suggests a position specific effect caused by the vibration stimulation.

Our experimental setup was carefully designed to minimize the impact of other possible factors for this phenomenon such as a change in intensity of the electrical stimulus or displacement of the recording electrodes.

### 4.1. Possible Mechanisms for Vibration-Induced Reduction of Digital SNAP

#### 4.1.1. Hyperpolarization

A prolonged high-frequency impulse train may hyperpolarize the afferent axons, thereby inhibiting the impulse propagation, which in turn may cause SNAP reduction [7]. This seems unlikely considering an approximately 40% increase in threshold attributable to this phenomenon. With the use of 100% supramaximal intensity, vibration-induced SNAP reduction remained the same, which, otherwise, would have activated the hyperpolarized axons. Second, hyperpolarization that is sufficient to produce a significant SNAP depression would have increased its onset latency, a finding not seen in our experiments [8]. Third, hyperpolarization develops slowly after the application of stimulation and wears off gradually over many minutes after its cessation [9, 10]. This stands in contrast to the present findings, where vibration caused immediate suppression of SNAPs, which then recovered to the baseline level as soon as the stimulation ceased.

#### 4.1.2. Collision and “Line Busy” Phenomenon

The magnitude of the size reduction of digital SNAP shown in the present study implies that vibration should have activated mechanoreceptors wildly, and the vibration-induced afferent volley should have come from multiple types of sensitive mechanoreceptors. It is likely that the reduction in the amplitude of the SNAP reflects spike collision between the vibratory evoked depolarization and the electrically evoked spike. The “line busy” hypothesis implies the occurrence of an afferent spikes and associated after potential followed by the absolutely refractory, which prevents generation of action potentials, as suggested by Hagbarth [11]. Consistent with single nerve fiber studies in animals [12], microneurographic recordings in humans demonstrated the absolute refractory periods of the distal median nerve sensory axons to range from 0.7 to 4.5 ms (mean: 2.1 ± 0.9 ms) for all the afferent fibers with no difference between the rapid and slow adapting afferents [4, 13]. Paired stimulus technique [14] also yielded the absolute refractory period of 0.7 ms for the human digital nerveSNAPs [15]. Maintaining the absolute refractory periods for all afferents would require the different types of A*β* fibers to discharge at approximately 222 to 1,428 Hz or above.

It has been well established that in studies such as by Muniak et al. [16] that low-frequency vibratory stimuli (e.g., 20 Hz at amplitude of 50 *μ*m) activate all types of hand mechanoreceptors and evoke multiple spikes per cycle. Nevertheless, there are no studies which provide direct evidences of the very-high-frequency tonic discharges of A*β* afferents during vibration to corroborate the hypotheses of “line busy” effects.

Focal vibratory stimuli have been used in neurorehabilitation including the neurological diseases or disorders like stroke, spinal cord injury, multiple sclerosis, Parkinson's disease, and dystonia. Focal vibration stimulated the proprioceptive system to obtain an efficient motor control in functional activities [2]. Our study demonstrated in vivo that the A*β* afferent fibers (proprioceptive system) were stimulated by vibratory stimulation and coursed the reduction of the responsiveness of A*β* afferent fibers to the electrical stimulation.

In conclusion, the remarkable drop of the SNAP size during acute exposure to vibratory stimulation reflects the significant reduction of the responsiveness of A*β* afferents to electrical stimulation. These changes of electrophysiological features of SNAP deserve further investigation in the study of the effects of focal vibration in neurorehabilitation.

## Figures and Tables

**Table 1:** Measurements of digit 3 SNAP.

*N* = 19	Amplitude (*μ*V)		*P*
	Mean	Reduction (%)	
Baseline	54.5 ± 6.9		<0.05
Vib MCPJ 3	22.3 ± 5.1	58.9 ± 8.6	
Vib MCPJ 5 (*N* = 7)	40.0 ± 3.3	27.5 ± 3.2	<0.05
After vibration	54.3 ± 6.7		

Vib MCPJ 3: vibration at MCPJ3; Vib MCPJ 5: vibration at MCPJ 5.

## Data Availability

Datasets analyzed during the current study are available from the corresponding author on reasonable request.

## Supplementary Materials

Supplementary 1 Supplementary Figure 1 Baseline: the amplitude of recording of Sensory Nerve Action Potential (SNAP) of digit 3before vibration with electrical stimulation of the median nerve of the wrist. Vib MCPJ 3: the amplitude of recording of SNAP of digit 3 DURING vibration at 3rd metacarpal phalangeal joint (MCPJ) with electrical stimulation of the median nerve of the wrist. Vib MCPJ 5: the amplitude of recording of SNAP of digit 3 DURING vibration at 5th MCPJ with electrical stimulation of the median nerve of the wrist. Vib ceased: the amplitude of recording of SNAP of digit 3 after vibration ceased immediately with electrical stimulation of the median nerve of the wrist. There are significant statistical differences of digit 3 SNAP between vibration at MCPJ 3 and baseline (^∗∗^, *P* < 0.05), vibration at MCPJ 5 and baseline (##, *P* < 0.05), vibration at MCPJ 3, and vibration at MCPJ 5 (^∗∗∗^, P < 0.05). There are no significant statistical differences of digit 3 SNAP between baseline and vibration ceased (^∗^, *P* > 0.05).

Supplementary 2 Supplementary Table 1 Digit 3 SNAP amplitude (*μ*V) before, during, and after vibration of MCPJ 3 and MCPJ 5.
