# Supplementary material for: Focal Vibration Alters Human Digital Sensory Nerve Action Potentials: A Pilot Study
Source: Neural Plast. 2021 Mar 3;2021:8819169. doi: 10.1155/2021/8819169 (PMC7949868; doi:10.1155/2021/8819169)
Supplement: Supplementary 1 — Supplementary Figure 1 Baseline: the amplitude of recording of Sensory Nerve Action Potential (SNAP) of digit 3before vibration with electrical stimulation of the median nerve of the wrist. Vib MCPJ 3: the amplitude of recording of SNAP of digit 3 DURING vibration at 3rd metacarpal phalangeal joint (MCPJ) with electrical stimulation of the median nerve of the wrist. Vib MCPJ 5: the amplitude of recording of SNAP of digit 3 DURING vibration at 5th MCPJ with electrical stimulation of the median nerve of the wrist. Vib ceased: the amplitude of recording of SNAP of digit 3 after vibration ceased immediately with electrical stimulation of the median nerve of the wrist. There are significant statistical differences of digit 3 SNAP between vibration at MCPJ 3 and baseline (∗∗, P < 0.05), vibration at MCPJ 5 and baseline (##, P < 0.05), vibration at MCPJ 3, and vibration at MCPJ 5 (∗∗∗, P < 0.05). There are no significant statistical differences of digit 3 SNAP between baseline and vibration ceased (∗, P > 0.05). [file 8819169.f1.docx]

**Supplementary FIGURE 1**. Baseline: the amplitude of recording of Sensory Nerve Action Potential (SNAP) of digit 3before vibration with electrical stimulation of the median nerve of the wrist. Vib MCPJ 3: the amplitude of recording of SNAP of digit 3 DURING vibration at 3rdmetacarpal phalangeal joint(MCPJ)with electrical stimulation of the median nerve of the wrist. Vib MCPJ 5: the amplitude of recording of SNAP of digit 3 DURING vibration at5thMCPJ with electrical stimulation of the median nerve of the wrist. Vib ceased: the amplitude of recording of SNAP of digit 3 after vibration ceased immediately with electrical stimulation of the median nerve of the wrist.There are significant statistical differences of digit 3 SNAP between vibration at MCPJ 3 and baseline(**,P＜0.05),vibration at MCPJ 5 and baseline(##,P＜0.05),vibration at MCPJ 3 and vibration at MCPJ 5(***,P＜0.05). There are no significant statistical differences of digit 3 SNAP between baseline and vibration ceased(*,P＞0.05).
