# Supplementary material for: Focal Vibration Alters Human Digital Sensory Nerve Action Potentials: A Pilot Study
Source: Neural Plast. 2021 Mar 3;2021:8819169. doi: 10.1155/2021/8819169 (PMC7949868; doi:10.1155/2021/8819169)
Supplement: Supplementary 2 — Supplementary Table 1 Digit 3 SNAP amplitude (μV) before, during, and after vibration of MCPJ 3 and MCPJ 5. [file 8819169.f2.docx]

| **Supplementary Table 1:** Digit 3 SNAP Amplitude (μV) before, during and after vibration of MCPJ 3 and MCPJ 5 | | | | | | |
| --- | --- | --- | --- | --- | --- | --- |
| Subject | Before(μV) | During MCPJ 3  (μV) | After Vibration  (μV) | Reduction MCPJ 3  (%) | During MCPJ 5  (μV) | Reduction  MCPJ 5  (%) |
| 1 | 50.4 | 21.9 | 50 | 56.5 | 38.1 | 24.4 |
| 2 | 52.5 | 24.4 | 52.1 | 53.5 | 37.6 | 28.4 |
| 3 | 58.5 | 21.7 | 58.1 | 62.9 | 44.5 | 31.5 |
| 4 | 54.3 | 21.6 | 54 | 60.2 |  |  |
| 5 | 46.6 | 18.3 | 46.8 | 60.7 | 35.6 | 23.6 |
| 6 | 46.2 | 15 | 46.2 | 67.5 |  |  |
| 7 | 63.9 | 24.2 | 63.2 | 62.1 | 43.8 | 31.5 |
| 8 | 61.7 | 22 | 61.2 | 64.3 |  |  |
| 9 | 53.2 | 19 | 52.5 | 64.3 | 39.8 | 25.2 |
| 10 | 59.2 | 18.3 | 58.1 | 69.1 |  |  |
| 11 | 56.1 | 32 | 55.7 | 43.0 |  |  |
| 12 | 56.3 | 30 | 56.2 | 46.7 |  |  |
| 13 | 46 | 24 | 46.1 | 47.8 |  |  |
| 14 | 52.3 | 24.9 | 53 | 52.4 |  |  |
| 15 | 45.1 | 13.7 | 46.1 | 69.6 |  |  |
| 16 | 68 | 22.2 | 67.5 | 67.4 |  |  |
| 17 | 63.6 | 21.7 | 64.4 | 65.9 |  |  |
| 18 | 56.5 | 32 | 56.1 | 43.4 | 40.7 | 28.00 |
| 19 | 44.7 | 16.8 | 44.9 | 62.4 |  |  |
| Mean | 54.5±6.9 | 22.3±5.1 | 54.3±6.7 | 58.9±8.6 | 40.0±3.3 | 27.5±3.2 |

MCPJ 3: vibration at the 3^rd^ metacarpolphalangeal joint;

MCPJ 5, vibration at the 5^th^ metacarpolphalangeal joint.

Before: Amplitude of Digit 3 SNAP before vibration (baseline).

During MCPJ 3:Amplitude of Digit 3 SNAP during vibration at MCPJ 3.

After Vibration :Amplitude of Digit 3 SNAP after vibration (vibration ceased) at MCPJ 3.

Reduction MCPJ 3: percentage of the reduction of amplitude of Digit 3 SNAP during vibration at MCPJ 3 compare with baseline.

During MCPJ 5:Amplitude of Digit 3 SNAP during vibration at MCPJ 5.

Reduction MCPJ 5: percentage of the reduction of amplitude of Digit 3 SNAP during vibration at MCPJ 5 compare with baseline.
